# Prognostic role of docetaxel-induced suppression of free testosterone serum levels in metastatic prostate cancer patients

**DOI:** 10.1038/s41598-021-95874-y

**Published:** 2021-08-12

**Authors:** Paula Kappler, Michael A. Morgan, Philipp Ivanyi, Stefan J. Brunotte, Arnold Ganser, Christoph W. M. Reuter

**Affiliations:** 1 Department of Hematology, Hemostaseology, Oncology, and Stem Cell Transplantation, Hannover Medical School, Carl-Neuberg-Str. 1, 30625 Hannover, Germany; 2 Institute of Experimental Hematology, Hannover Medical School, Carl-Neuberg-Str. 1, 30625 Hannover, Germany

**Keywords:** Cancer, Prostate cancer

## Abstract

To date, only few data concerning the biologically active, free form of testosterone (FT) are available in metastatic prostate cancer (mPC) and the impact of FT on disease, therapy and outcome is largely unknown. We retrospectively studied the effect of docetaxel on FT and total testosterone (TT) serum levels in 67 mPC patients monitored between April 2008 and November 2020. FT and TT levels were measured before and weekly during therapy. The primary endpoint was overall survival (OS). Secondary endpoints were prostate-specific antigen response and radiographic response (PSAR, RR), progression-free survival (PFS), FT/TT levels and safety. Median FT and TT serum levels were completely suppressed to below the detection limit during docetaxel treatment (FT: from 0.32 to < 0.18 pg/mL and TT: from 0.12 to < 0.05 ng/mL, respectively). Multivariate Cox regression analyses identified requirement of non-narcotics, PSAR, complete FT suppression and FT nadir values < 0.18 pg/mL as independent parameters for PFS. Prior androgen-receptor targeted therapy (ART), soft tissue metastasis and complete FT suppression were independent prognostic factors for OS. FT was not predictive for treatment outcome in mPC patients with a history of ART.

## Introduction

Prostate cancer (PC) growth and progression is androgen-dependent and androgen deprivation therapy (ADT) is an effective strategy to manage advanced disease^1–3^. Historically, suppression of total testosterone (TT) to a level lower than 50 ng/dL (< 1.7 nmol/L) has been defined as castration. However, a testosterone suppression target of less than 20 ng/dL improves patient survival and delays tumor progression^1,4^. Androgen receptor (AR) targeted therapy (ART) in combination with ADT led to testosterone suppression to near zero and further improved patient survival^1^. Castration-resistance occurs despite castrate testosterone levels through reactivation of AR pathways from multiple mechanisms. Furthermore, neuroendocrine transdifferentiation may also occur in PC and lead to castration resistance, which results in shorter progression-free (PFS) and overall survival (OS)^1^. Due to its critical role, testosterone levels should be monitored frequently during therapy^1^.

Ryan et al. found that docetaxel therapy significantly reduced androgen levels, including total testosterone (TT), with increased OS (26.3 vs. 20.9 months), with better outcome in patients with high versus low reduction of androgen levels, respectively^5^. Total serum androgens (TT, androstenedione, DHEAS) were described as important biomarkers in PC treatment and may be useful in risk stratification in future studies^5,6^.

Plasma testosterone (total testosterone, TT) circulates specifically bound to sex hormone-binding globulin (SHBG) (50–70%), nonspecifically bound to albumin (20–30%) or other proteins (4%) and unbound (1–3%), or free (FT)^7^. Bioavailable testosterone (BT) is calculated by adding FT and albumin-bound testosterone levels^7,8^. Quantification of FT is an efficient method to evaluate BT^8^. Although FT is a key target in the treatment of advanced prostate cancer, the effects that FT may have on PC treatment and outcome are largely unknown^9^. The castration level of FT was established to be < 1.7 pg/mL (< 5.9 pmol/L)^8^. Von Klot et al. identified FT < 0.5 pg/mL as a prognostic marker in second-line therapy, which was associated with improved cancer-related survival (43.6 vs. 17.3 months)^10^.

Docetaxel seems to be less efficient after treatment with ART, with a reduced PSAR rate after ART (median PSAR group 1: docetaxel → cabazitaxel → ART: 59.8%; group 2: docetaxel → ART → cabazitaxel: 64.3%; group 3: ART → docetaxel → cabazitaxel: 44.0%; *p* = 0.021) and significantly shorter PFS and OS (median radiographic PFS for group 1: 26.9 (14.8-NR), group 2: 11.0 (9.5–12.9) and group 3: 6.6 (5.0–10.2), *p* < 0.001; median OS for group 1: 34.8 (32.4–41.5) months; group 2: 35.8 833.9–38.4) months; group 3: 28.9 (23.3–35.9) months; *p* = 0.007)^11^. A certain cross-resistance of docetaxel and ART was discussed as a potential explanation for these findings^11,12^. Studies suggest that docetaxel influences androgen receptor signaling, while testosterone impairs cellular uptake of docetaxel and inhibits stabilization of microtubules. This led to the proposal that testosterone levels should be decreased during chemotherapy with docetaxel^13,14^.

Even in the castration-resistant setting, PC tumors still rely on androgen signaling^15^. Hence, the goal of this study was to investigate the effect of docetaxel on FT and TT at different stages of metastatic PC (e.g. castration-naïve (mCNPC, group 1), castration-resistant (mCRPC, group 2) and castration-resistant patients with prior ART (mCRPC-ART, group 3)).

## Methods

### Patients

For this translational biomarker study, data from patients with mPC treated with docetaxel at Hannover Medical School from April 2008 to November 2020 were retrospectively analyzed and followed up until death or until December 2020. Eligibility criteria were a histologically confirmed mPC and disease progression according to the PCWG2/3 criteria^4,16^. Patients who received at least two cycles of docetaxel were included. All data were collected following patient informed consent, in accordance with the principles of the Declaration of Helsinki and Good Clinical Practice guidelines, and with the Hannover Medical School institutional review board approval (13th August 2008). Patients were stratified into three subgroups: (1) castration-naïve disease (mCNPC), (2) castration-resistant disease (mCRPC) and (3) mCRPC patients with a history of ART (mCRPC-ART).

### Treatment plan

At baseline, medical history and physical examination were performed, including an initial staging. Patients received 75 mg/m^2^ of docetaxel intravenously every 3 weeks (q3w), 50 mg/m^2^ every 2 weeks (q2w) or 30–35 mg/m^2^ weekly on days 1, 8 and 15 (q1w). Concomitant use of dexamethasone and oral prednisone (5 mg) twice a day were part of the regimen. ADT was continued throughout therapy. Morning FT and TT (8–11 am) were obtained before and weekly during treatment (on average 28 samples per patient) using an enzyme immunoassay (ELISA from IBL, International GmbH, Hamburg, Germany) and a direct, competitive, chemiluminescence immunoassay (CLIA) (LIAISON^®^ Testosterone Assay, Diasorin S.p.A., Saluggia, Italy). PSA levels, carcinoembryonic antigen (CEA) levels, neuroendocrine tumor markers (neuron-specific enolase (NSE), chromogranin A (CgA)), lactate dehydrogenase (LDH), alkaline phosphatase (AP), hemoglobin and analgesic requirement were parameters at baseline to possibly predict OS^16^. Patients were assessed according to PCWG2/3 criteria and RECIST 1.1 by CT and bone scan every three months or if tumor progression was suspected^4,17,18^. Pain and use of pain medications were monitored by clinician interview. Toxicity was graded according to the Common Terminology Criteria for Adverse Events v3.0 (CTCAE).

### Data analysis

The primary study end point was OS, defined by the initiation of docetaxel therapy until death. Secondary endpoints were PFS, defined as the time between the start of docetaxel therapy until progression according to PCWG2/3 criteria^4,16^, PSA response (PSAR), which is defined by a decline of > 50% from baseline, and FT reduction of 100% from baseline and safety. Radiographic response (RR) was evaluated according to RECIST 1.1.^17^. Follow-up data were collected throughout December 2020.

Statistical analyses were performed using SPSS statistics v26.0. Categorical variables were summarized, numeric variables were analyzed in median and range. Logistic regression was used to estimate the prognostic significance of FT suppression in predicting ≥ 50% decline in PSA from baseline. Cox proportional hazards regression modeling was used to determine the prognostic significance of baseline characteristics on PFS and OS. Chi-square tests and t-tests were applied to estimate *p *values of variables at baseline. Uni- and multivariate Cox regression analyses were used to demonstrate the impact of covariates on PFS and OS. Non-proportionality was assessed by plotting the Kaplan–Meier survival distribution as a function of the survival time for each level of the covariate and plotting the function log(-log(survival probability)) as a function of the log survival time^19^. Additionally, extended Cox modelling with time-by-covariates and conditional landmark analyses were used to remove potential guarantee-time bias, specifically the time-window bias, which is introduced because of differential exposure opportunity time windows between subjects^20^. Only *p* values of < 0.05 were considered statistically significant for all comparisons.

## Results

### Patient characteristics

A total of 67 patients with a histologically confirmed metastatic adenocarcinoma of the prostate were analyzed. Seven patients were castration-naïve (mCNPC, group 1), 26 patients were castration-resistant after ADT (mCRPC, group 2) and 34 patients were castration resistant after ADT plus ART (mCRPC-ART, group 3). The median age at time of diagnosis was 69 years (Table 1).

**Table 1:** Patient characteristics, prior treatments and laboratory values at baseline before start of docetaxel chemotherapy for 67 patients.

	All n = 67	Group 1 mCNPC n = 7	Group 2 mCRPC n = 26	Group 3 mCRPC-ART n = 34	*p* value
Age, median	69	69	68.5	69	
**ECOG, no. (%)**					
0	22 (32.8)	3 (42.9)	12 (46.2)	7 (20.6)	
1	31 (46.3)	4 (57.1)	11 (42.3)	16 (47.1)	
2	10 (14.9)	0	3 (11.5)	7 (20.6)	
3	4 (6)	0	0	4 (11.8)	0.009
Gleason score 8–10, no. (%)	36 (53.7)	2 (28.6)	15 (57.7)	19 (55.9)	
BMI	26.2	24.7	27.8	25.3	0.025
**Metastases**					
*No. of organs, no. (%)*					
1	28 (41.8)	2 (28.6)	13 (50)	13 (38.2)	
2	24 (35.8)	3 (42.8)	5 (19.2)	16 (47.1)	
≥ 3	15 (22.4)	2 (28.6)	8 (30.8)	5 (14.7)	
Bone	64 (95.5)	7 (100)	25 (96.2)	32 (94.1)	
Soft tissue	42 (62.7)	5 (71.4)	14 (53.9)	23 (67.7)	
Lymph nodes	34 (50.8)	5 (71.4)	12 (46.2)	17 (50)	
Lungs	11 (16.4)	1 (14.3)	6 (23.1)	4 (11.8)	
Liver	11 (16.4)	0	5 (19.2)	6 (17.7)	
Brain	2 (3)	0	2 (7.7)	0	
Cancer pain, no. (%)	38 (56.7)	6 (85.7)	14 (53.9)	18 (52.9)	
Non-narcotics required, no. (%)	26 (38.8)	4 (57.1)	8 (30.8)	14 (41.2)	
Narcotics required, no. (%)	23 (34.3)	2 (28.6)	7 (26.9)	14 (41.2)	
**Prior treatment**					
*Local therapy*					
Radical prostatectomy, no. (%)	24 (35.8)	0	13 (50)	11 (32.4)	0.015
TUR-prostate, no. (%)	13 (19.4)	0	7 (26.9)	6 (17.7)	
**Radiotherapy**					
RTX prostate, no. (%)	19 (28.4)	0	8 (30.8)	11 (32.4)	
RTX bone, no. (%)	34 (50.8)	3 (42.9)	14 (53.9)	17 (50)	
RTX soft tissue, no. (%)	10 (14.9)	0	4 (15.4)	6 (17.7)	
**Radiopharmaceuticals**					
Alpharadin, no. (%)	3 (4.5)	0	0	3 (8.8)	
PSMA ligands, no. (%)	1 (1.5)	0	0	1 (2.9)	
Duration of ADT in months	17 (0–108)	0	22.5	25	0.008
**ART**					
Abiraterone, no. (%)	29 (43.3)	0	0	29 (85.3)	< 0.001
Enzalutamide, no. (%)	20 (29.9)	0	0	20 (58.8)	0.009
Ketoconazole, no. (%)	2 (3)	0	1 (3.9)	1 (2.9)	
Estramustine, no. (%)	5 (7.5)	0	4 (15.4)	1 (2.9)	
Prior docetaxel, no. (%)	18 (26.8)	0	5 (19.2)	13 (38.2)	
**Laboratory at baseline**					
PSA (μg/L), median (range)	129.7 (4.26–6695)	737.9	102.85	128.3	0.001
FT (pg/mL), median (range)	0.315 (0.18–15.3)	1.92	1.14	0.18	< 0.001
TT (ng/mL), median (range)	0.12 (0.05–4.86)	2.4	0.19	0.12	< 0.001
Hb (g/dL), median (range)	11.5 (7.4–15.6)	10.2	12.1	11.5	0.029
AP (U/L), median (range)	151 (44–4834)	307	122	146	
> ULN, no. (%)	36 (53.7)	7 (100)	12 (46.2)	17 (50)	
> 2xULN, no. (%)	23 (34.3)	5 (71.4)	7 (26.9)	11 (32.4)	
LDH (U/L), median (range)	285 (158–1266)	422	278	276.5	
> ULN, no. (%)	41 (61.2)	6 (85.7)	16 (61.5)	19 (55.9)	
> 2 × ULN, no. (%)	13 (19.4)	1 (14.3)	6 (23.1)	6 (17.7)	
NSE (μg/L), median (range)	21 (11–56)	22.5	20	23	
> ULN, no. (%)	40 (59.7)	5 (71.4)	13 (50)	22 (64.7)	
CgA (μg/L), median (range)	149.5 (2–664)	177	90	189	
> ULN, no. (%)	42 (62.7)	6 (85.7)	13 (50)	23 (67.7)	
CgA/NSE > ULN, no. (%)	49 (73.1)	7 (100)	17 (65.4)	26 (76.5)	
CgA and NSE > ULN, no (%)	31 (61.2)	4 (57.1)	9 (34.6)	19 (55.9)	0.018
NLR, median	5.8	5.3	4.9	6.9	

*ECOG* Eastern cooperative oncology group, *BMI* body mass index, *TUR-prostate* transurethral resection of the prostate, *RTX* radiation therapy, *PSMA* prostate-specific membrane antigen, *ADT* androgen-depleting therapy, *ART* androgen receptor targeted therapy, *PSA* prostate-specific antigen, *FT* free testosterone, *TT* total testosterone, *Hb* hemoglobin, *AP* alkaline phosphatase, *ULN* upper limit of normal, *LDH* lactate dehydrogenase, *NSE* neuron-specific enolase, *CgA* chromogranin A, *NLR* neutrophil to lymphocyte ratio.

*p* values were estimated by applying t-tests to evaluate significant differences between the groups at baseline.

At baseline, FT levels below the detection limit were more common in group 3 than in groups 1 and 2 (*p* < 0.001). PSA, FT and TT were higher in group 1 than in groups 2 and 3, whereas hemoglobin was lower. Overall, 95.5% patients had bone metastases and 62.7% had soft tissue metastases. A median of two organs were involved by metastatic disease. All other characteristics were well-balanced (Table 1).

### Effects of docetaxel chemotherapy on TT and FT

**Figure 1:**
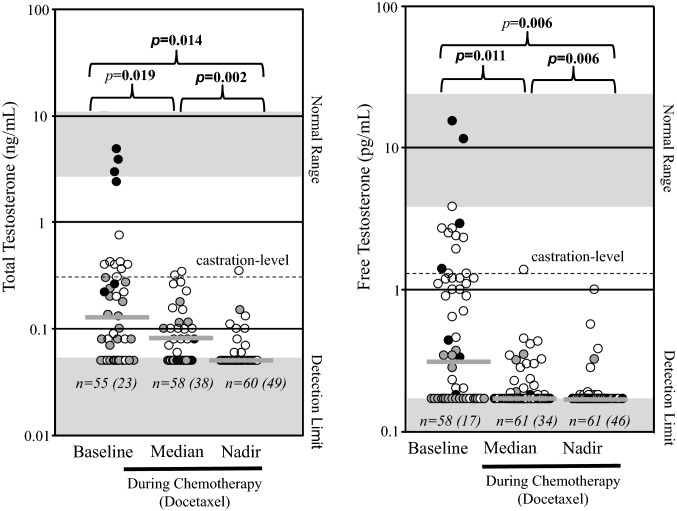
Distribution of baseline, median and nadir values of total testosterone (TT) and free testosterone (FT) levels. Castration level for TT is defined as 0.5 ng/mL^4^ and for FT as 1.7 pg/mL^9^. Numbers in brackets demonstrate number of patients with testosterone levels below detection limit. Black dots: mCNPC patients, grey dots: mCRPC patients, white dots: mCRPC-ART patients. The grey bars depict the median values of each group. The discrepancy in sample numbers in the groups is due to missing values in the database.

**Figure 2:**
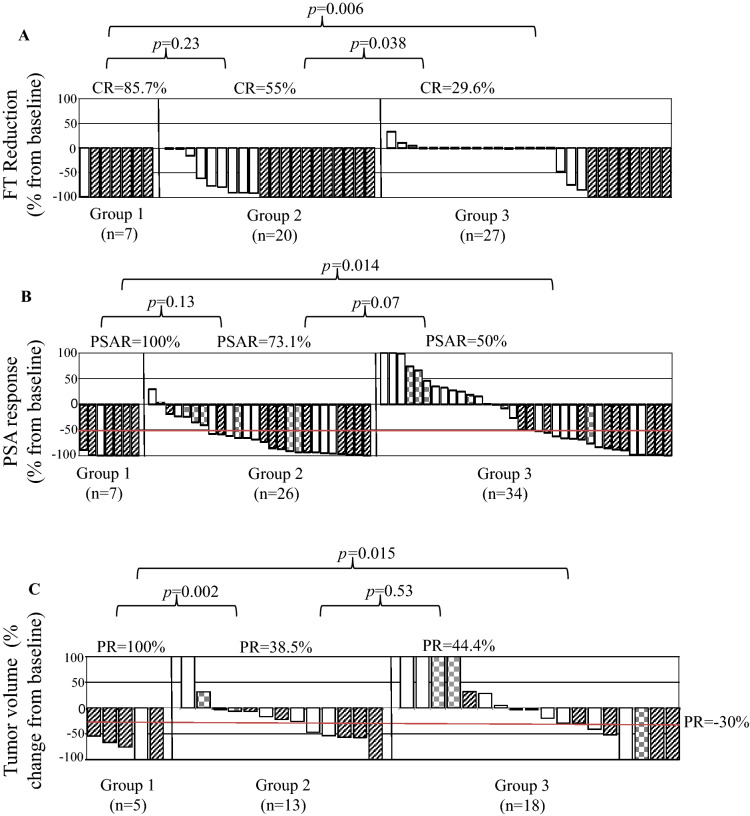
Response to docetaxel. (**A**) Free testosterone (FT) reduction, complete reduction (CR) is marked striped in every waterfall plot. White columns show data of patients with no CR and squared columns show missing values. (**B**) PSA response (PSAR) defined as decrease of 50% from baseline according to PCWG2/3 criteria^4,17^ during therapy is shown in the second plot. (**C**) Shrinkage of soft tissue tumor metastases is given as percent from baseline according to RECIST 1.1 (response evaluation criteria in solid tumors)^18^.

In the overall study population, serum levels of TT were reduced from a median of 0.12 ng/mL at baseline to non-detectable levels (< 0.05 ng/mL) at nadir (*p* = 0.014) and FT levels were reduced from 0.32 pg/mL at baseline to non-detectable levels at nadir (< 0.18 pg/mL) during docetaxel chemotherapy (*p* = 0.006; Fig. 1). The rate of patients with TT levels under the detection limit (< 0.05 ng/mL) increased from 23/55 (41.8%) at baseline (all 23 receiving abiraterone at that time point) to 49/60 patients (81.7%) at nadir during chemotherapy with docetaxel (*p* = 0.014) (Fig. 1). Similarly, the rate of patients with FT levels under the detection limit (< 0.18 pg/mL) increased from 17/58 (29.3%) (all 17 receiving abiraterone at that time point) at baseline to 46/61 (75.4%) at nadir during chemotherapy with docetaxel (*p* = 0.006) (Fig. 1). Complete FT suppression below the detection limit (< 0.18 pg/mL) was observed in 6/7 patients (85.7%) of group 1 (mCNPC), 11/20 (55%) of group 2 (mCRPC) and 8/27 (29.6%) of group 3 (mCRPC-ART) (Fig. 2A). FT suppression below the detection limit (< 0.18 pg/mL) was significantly associated with PSA response (*p* = 0.008; odds ratio 0.111 95% CI 0.022–0.564) (Fig. 2B). FT suppression was also associated with radiographic response (RECIST) (*p* = 0.051; odds ratio 0.218 95% CI 0.047–1.005; chi-square test *p* = 0.006) (Fig. 2C). Partial remission was observed in 5/5 patients of group 1 (mCNPC), 5/13 patients (38.5%) of group 2 (mCRPC) and 8/18 patients (44.4%) of group 3 (mCRPC-ART) (Fig. 2C). The median time to FT nadir was 14 days (95% CI 12.8/28.5, n = 37).

### Clinical outcome

Patients underwent a median of eight cycles of docetaxel (range 2–108) and were treated with a median cumulative dosage of 663 mg/m^2^ (range 100–8100 mg/m^2^). Group 1 (mCNPC) and group 2 (mCRPC) received a higher cumulative docetaxel dose than group 3 (mCRPC-ART) (1275 mg/m^2^ vs. 450 mg/m^2^, *p* < 0.001, 750 mg/m^2^ vs. 450 mg/m^2^, *p* = 0.004, respectively, Table 2). The overall PSAR rate (≥ 50%) was 43/67 (64.2%): 7/7 (100%) in group 1 (mCNPC), 19/26 (73.1%) in group 2 (mCRPC) and 17/34 (50%) in group 3 (mCRPC-ART) (*p* = 0.13 and *p* = 0.014, respectively; Fig. 2B, Table 2). The overall median PSA reduction was − 66.5% (range + 100 to − 99.9%): a median of − 99.7% in group 1; − 70.9% in group 2 and − 50% in group 3, respectively (*p* = 0.007) (Table 2). The median time to PSAR (≥ 50%) was 44 days (range 7–329).

**Table 2:** Treatment outcome, survival and clinical responses.

	All n = 67	Group 1 mCNPC n = 7	Group 2 mCRPC n = 26	Group 3 mCRPC-ART n = 34	*p* value
**Initial dosage, no. (%)**					
75 mg/m^2^	45 (67.2)	6 (85.7)	24 (92.3)	15 (44.1)	
50 mg/m^2^	15 (22.4)	0	1 (3.9)	14 (41.2)	
35 mg/m^2^	7 (10.5)	1 (14.3)	1 (3.9)	5 (14.7)	
Dose reduction, no. (%)	12 (17.9)	1 (14.3)	5 (19.2)	6 (17.7)	
No. of cycles, median (range)	8 (2–108)	17	12	6.5	
Cumulative dose, median (range)	663 (100–8100)	1275	750	450	< 0.001
Duration of CTX in months, median (range)	7 (1–78)	15	10.5	6	0.004
OS in months (95% CI)	25.7 (18.9–32.5)	33.7 (30.9–36.5)	27.4 (14.2–40.6)	16.8 (16.2–17.4)	0.002
PFS in months (95% CI)	7.8 (3.9–11.7)	15.4 (14.7–16.2)	11.3 (6.3–16.4)	5.5 (2.5–8.6)	0.018
PSA response, no. (%)	43 (64.2)	7 (100)	19 (73.1)	17 (50)	0.014
PSA reduction (%), median (range)	− 66.5 (+ 100 − (− 99.9))	− 99.7	− 70.9	− 50	0.007
Time to PSA response in days, median (range)	44 (7–329)	21	44	48	0.031
FT median (pg/mL), median (range)	0.18 (0.18–1.85)	0.18	0.21	0.18	< 0.001
FT nadir (pg/mL), median (range)	0.18 (0.18–1)	0.18	0.18	0.18	0.001
FT reduction (%), median (range)	− 91.3 (+ 33 − (− 100))	− 100	− 100	0	
TT median (ng/mL), median (range)	0.08 (0.05–0.34)	0.05	0.1	0.05	0.03
TT nadir (ng/mL), median (range)	0.05 (0.05–0.35)	0.05	0.05	0.05	
TT reduction (%) median (range)	− 62.5 (+ 120 − (− 100))	− 100	− 43.8	0	0.043
**Radiographic response (bone), no. (%)**	n = 64	n = 7	n = 25	n = 32	
Improved	10 (15.6)	3 (42.9)	2 (8)	5 (16)	0.034
Mixed	4 (6.3)	0	2 (8)	2 (6.3)	
Stable	29 (45.3)	4 (57.1)	12 (48)	13 (40.6)	
Progression	13 (20.3)	0	7 (28)	6 (18.8)	
			NA: 2 (8)	NA: 6 (18.8)	
**(Soft tissue), no. (%)**	n = 42	n = 5	n = 14	n = 23	
RECIST (%), median	− 28	− 75.8	− 21.9	− 10	0.002
PR	18 (42.9)	5 (100)	5 (35.7)	8 (34.8)	0.017
SD	9 (21.4)	0	5 (35.7)	4 (17.4)	
PD	11 (26.2)	0	3 (21.4)	6 (26.1)	
	NA: 6 (2.6)		NA: 1 (7.1)	NA: 5 (21.7)	
Blood transfusion, no. (%)	22 (32.8)	2 (28.6)	7 (26.9)	13 (38.2)	
G-CSF, no. (%)	9 (13.4)	2 (28.6)	2 (7.7)	5 (14.7)	
**Adverse events, no. (%)**					
Fatigue	47 (70.2)	6 (85.7)	17 (65.4)	24 (70.6)	
Dyspnea	24 (35.8)	0	8 (30.8)	16 (47.1)	0.017
Nausea	24 (35.8)	2 (28.6)	8 (30.8)	14 (41.2)	
Diarrhea	9 (13.4)	0	1 (3.9)	8 (23.5)	0.041
Constipation	7 (10.5)	1 (14.3)	3 (11.5)	3 (8.8)	
Infection	16 (23.9)	2 (28.6)	6 (23.1)	8 (23.5)	
PNP	14 (20.9)	0	9 (34.6)	5 (14.7)	0.042
Nail changes	4 (6)	0	4 (15.4)	0	0.012
**Hemtatological toxicities, no. (%)**					
Anemia grade 1 + 2	49 (73.1)	7 (100)	20 (76.9)	22 (64.7)	
Anemia grade 3 + 4	6 (9)	0	1 (3.9)	5 (14.7)	
Leukopenia grade 1 + 2	27 (40.3)	3 (42.9)	10 (38.5)	14 (41.2)	
Leukopenia grade 3 + 4	18 (26.9)	3 (42.9)	8 (30.8)	7 (20.6)	
Neutropenia grade 1 + 2	11 (16.4)	0	4 (15.4)	7 (20.6)	
Neutropenia grade 3 + 4	22 (32.8)	4 (57.1)	11 (42.3)	7 (20.6)	0.047*
Thrombopenia grade 1 + 2	22 (32.8)	4 (57.1)	9 (34.6)	9 (26.5)	
Thrombopenia grade 3 + 4	3 (4.5)	0	2 (7.7)	1 (2.9)	

*CTX* chemotherapy, *CI* confidence interval, *OS* overall survival, *PFS* progression-free survival, *PSA* prostate-specific antigen, *FT* free testosterone, *TT* total testosterone, *NA* not available, *RECIST* response evaluation criteria in solid tumors, *PR* partial remission, *SD* stable disease, *PD* progressive disease, *G-CSF* granulocyte-colony stimulating factor.

*p* values were estimated by using t-tests and chi-square-test*.

There was a higher rate of improvement of bone lesions in group 1 than group 2 (42.9% vs. 7.7%, *p* = 0.034, Table 2). The shrinkage of soft tissue metastases objectified by RECIST 1.1 was significantly higher in group 1 than group 2 (− 75.8% vs. − 21.9, *p* = 0.002; Fig. 2C and Table 2). The rate of partial remission was also significantly higher in group 1 than in groups 2 and 3 (71.4% vs. 19.2%, *p* = 0.017 and 71.4% vs. 23.5%, *p* = 0.02, respectively).

**Figure 3:**
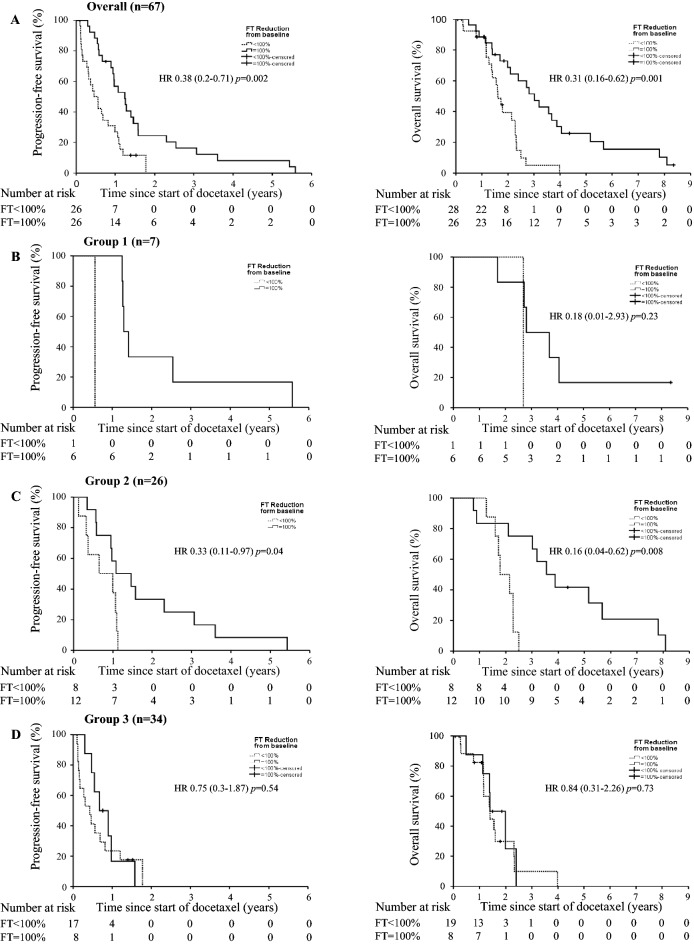
Kaplan–Meier curves for progression-free survival and overall survival for patients with free testosterone (FT) reduction of 100% (black line) and for patients with < 100% reduction of FT (dotted line).

Median OS was 25.7 months (95% CI 18.9–32.5) in all patients. mCNPC patients experienced a median OS of 33.7 months (95% CI 30.9–36.5), whereas patients in group 3 (mCRPC-ART) had a median OS of 16.8 months (95% CI 16.2–17.4; *p* = 0.002). Median PFS in all patients was 7.8 months (95% CI 3.9–11.7): 15.4 months (95% CI 14.7–16.2) for group 1, 11.3 months (95% CI 6.3–16.4) for group 2 and 5.5 months (95% CI 2.5–8.6) in group 3 (*p* = 0.033 and *p* = 0.018, respectively; Table 2, Fig. 3).

### Multivariate analyses

Univariate analyses of baseline parameters associated with OS are summarized in Table 3. In multivariate analyses with stepwise regression, only prior ART and the presence of soft tissue metastases (lymphatic, hepatic, pulmonary, brain) remained independent predictors of a shorter OS (Table 3).

**Table 3:** Baseline factors influencing OS and PFS: univariate and multivariate analysis.

	Univariate analysis HR (95% CI)	*p* value	Multivariate analysis HR (95% CI)	*p* value
**Overall survival**				
ECOG > 1	2.71 (1.37–5.34)	0.004		
Group (1–3)	2.27 (1.41–3.65)	0.001		
Prior ART overall	3.09 (1.69–5.67)	< 0.001	2.56 (1.28–5.11)	0.008
Response to ART	0.39 (0.15–0.99)	0.047		
Previous prednisone	2.8 (1.45–5.41)	0.002		
FT < detection level at baseline	2.05 (1.01–4.19)	0.048		
CRP > median	1.93 (1.01–3.68)	0.047		
NLR > 3	3.46 (1.35–8.88)	0.01		
Number of organs	1.73 (1.29–2.31)	< 0.001		
Soft tissue metastases	3.17 (1.67–6)	< 0.001	3.46 (1.81–6.6)	< 0.001
Lymphatic metastasis	2.38 (1.33–4.43)	0.003		
Hepatic metastasis	3.05 (1.43–6.48)	0.004		
PD soft tissue and bone at baseline	2.57 (1.39–4.74)	0.002		
Narcotics required	2.1 (1.16–3.8)	0.015		
Treatment-dependent parameters**				
FT reduction = 100%	0.31 (0.16–0.62)	0.001	0.28 (0.14–0.57)	< 0.001
FT median < 0.3 pg/mL	0.49 (0.24–1.0)	0.049		
**Progression-free survival**				
Group (1–3)	1.87 (1.24–2.8)	0.003		
Prior ART overall	2.14 (1.25–3.66)	0.006		
Response to ART	0.39 (0.17–0.92)	0.031		
Prior enzalutamide	2.11 (1.18–3.76)	0.012		
Prior abiraterone	1.75 (1.02–2.99)	0.043		
PD soft tissue and bone at baseline	1.85 (1.06–3.2)	0.029		
NLR > 3	4.87 (1.85–12.81)	0.001		
Soft tissue metastases	2.35 (1.28–4.32)	0.006		
Lymphatic metastasis	2.04 (1.16–3.6)	0.013		
Number of organs	1.47 (1.12–1.93)	0.006		
Non-narcotics required	1.93 (1.11–3.39)	0.021	2.72 (1.1–6.72)	0.03
Narcotics required	2.36 (1.3–4.3)	0.005		
**Treatment-dependent parameters ♦**				
PSA response	0.33 (0.19–0.58)	< 0.001	0.32 (0.15–0.66)	0.002
FT reduction = 100%	0.38 (0.2–0.71)	0.002	0.43 (0.22–0.84)	0.014
FT median < 0.2 pg/mL	0.47 (0.25–0.91)	0.024		
FT nadir < 0.18 pg/mL	0.46 (0.25–0.86)	0.014	0.39 (0.18–0.81)	0.011

*HR* hazard ratio, *CI* confidence interval, *ECOG* Eastern Cooperative Oncology Group, *ART* androgen receptor targeted therapy, *FT* free testosterone, *CRP* C-reactive protein, *NLR* neutrophile to lymphocyte ratio, *PD* progressive disease, *PSA* prostate-specific antigen, *FT* free testosterone.

** = time-dependent covariate analysis revealed for FT reduction = 100% and FT median < 0.3 pg/mL *p* > 0.05 for overall survival applying T_, ln(T_) and T ≥ 365 days.

**♦** = ln(T) revealed for PSA response *p* = 0.015, for the remaining time dependent parameters using T_,ln(T) and T ≥ 365 days there was no significant time-dependence.

In univariate and time-dependent covariate analyses (T_; ln(T_); T_ ≥ 365 days; data not shown) of treatment-dependent parameters (e. g. FT-, PSA-response), only FT median < 0.3 pg/mL and complete FT suppression (reduction of 100%) during docetaxel therapy were associated with a longer OS. In multivariate analysis, complete FT reduction (100%) remained an independent predictor for better OS (Table 3).

In addition to extended time-dependent Cox modelling, log(-log(survival probability)) and conditional landmark analyses were performed and demonstrated that the FT reduction = 100% during docetaxel therapy had no association with time regarding OS (data not shown).

In multivariate analysis of the baseline parameters, only the requirement of non-narcotics remained an independent predictor of a better PFS (Table 3). In univariate analysis of treatment-dependent parameters, PSAR, FT suppression (100%), FT median values < 0.2 pg/mL and FT nadir values < 0.18 pg/mL during docetaxel therapy were associated with a better PFS. In multivariate analysis with stepwise regression, only PSAR, FT reduction = 100% and FT nadir values < 0.18 pg/mL remained independent predictors of a better PFS. Applying extended time-dependent Cox modelling (T_; ln(T_); T_ ≥ 365 days), log(-log(survival probability)) and conditional landmark analyses demonstrated that these parameters had no association with time, with the exception of ln(T_) for PSAR, which revealed a significant time-dependence (*p* = 0.015, Table 3) (data not shown).

## Discussion

In this study, we demonstrate that TT and FT serum levels are reduced during docetaxel chemotherapy and that FT suppression under the detection limit (100%) resulted in better PFS and OS in mCNPC and mCRPC patients, but not in mCRPC patients with a history of ART. Interestingly, in contrast to TT, only FT was a significant predictor for PFS and OS, demonstrating a major biological role of FT for treatment outcome. mCRPC-ART patients had a significantly lower FT reduction rate due to low FT levels at baseline (8/27 vs. 6/7 and 11/20, respectively) and FT reduction was no longer a predictor for better PFS or OS (Table 2). mCRPC-ART patients experienced a lower PSAR and shorter PFS and OS. These results are consistent with several previous studies that showed decreased efficacy of docetaxel in PC patients with a history of ART^11,12,21,22^. Our data suggest that the worst clinical outcome of mCRPC-ART patients towards docetaxel is due to progressing castration resistance.

Multiple lines of evidence suggest that docetaxel and prednisone might directly interfere with testosterone biosynthesis and metabolism in mPC patients and contribute to this FT suppressing effect. Prednisone was demonstrated to lower serum TT, androstenedione, dehydroepiandrosterone (DHEA) and dehydroepiandrosterone sulfate (DHEAS) levels in some metastatic PC patients by suppressing the hypothalamic–pituitary–adrenal axis, but had no antitumor activity in mCRPC^23,24^. Consistent with this observation, a history of previous prednisone treatment had no effect in multivariate analyses on PFS or OS in our study.

The role of docetaxel in reducing testosterone levels is less clear, although serum androgens (TT, androstenedione and DHEA) decline during docetaxel treatment^5^. Docetaxel metabolism is largely catalyzed by CYP3A4^25^ and docetaxel was shown to induce CYP3A4, which is responsible for the greatest portion of testosterone 6β- and 16β-hydroxylation^26–28^. CYP3A4 induction may lower testosterone levels by inactivation through 6β- and 16β-hydroxylation. The effect of docetaxel on CYP17A1 is unclear^26–28^.

Franke et al. reported castration-dependent pharmacokinetics of docetaxel in PC patients. Docetaxel clearance was increased by approximately 100% in castrated men and was associated with a two-fold reduction in area under the curve, although hepatic activity of CYP3A4 was unchanged^29^. Conversely, castration-naïve patients were exposed to higher amounts of the drug, which was accompanied by more severe hematotoxicity^29^. This study also demonstrated that lower intracellular docetaxel levels caused by lower baseline levels of testosterone resulted in a lower response rate to treatment^29^. Consistent with these results, group 1 patients (mCNPC) had a significantly higher rate of grade 3 and 4 neutropenia compared to group 3 patients (mCRPC-ART) in our study (57.1% vs. 20.6%, *p* = 0.047; Table 2) and a significantly better clinical outcome (Figs. 2, 3, Table 2).

Ryan et al. showed that conversion from higher to lower androgen levels (e.g. above/below median) during docetaxel therapy contributed to superior survival as the reduction is the driving mechanism behind the clinical responses^5^. Consistent with these findings, 6/7 (85.7%) mCNPC patients in our study underwent a complete (= 100%) and one patient a nearly complete (99.3%) FT reduction and had a PSA response rate of 100%.

In recent years, several large phase 3 trials in patients with mCNPC (e.g. CHAARTED, STAMPEDE, GETUG-3, LATTITUDE, TITAN, PREVAIL) demonstrated that the addition of docetaxel and ART (abiraterone, apalutamide and enzalutamide) to ADT is associated with significantly improved PFS and/or OS compared to ADT alone^30^. Our data demonstrate that docetaxel therapy is associated with similarly low testosterone levels (FT + TT) as achieved by Abiraterone + ADT (Fig. 1, Tables 1, 2).

As with many retrospective analyses, the retrospective design of our study also has some limitations. For example, the patient population size is small, and although high numbers of FT and TT measurements were accomplished, these were not always assessed on a regular basis (e.g. weekly). Furthermore, progression was mainly due to PSA progression (e.g. PSA progression or radiographic progression, whichever presented first). Despite all of our efforts to address possible lead-time bias (e.g. use of extended time-dependent Cox modelling (T_; ln(T_); T_ ≥ 365 days), log(-log(survival probability)) and conditional landmark analyses), there is still the risk that our analyses are subject to lead-time bias, as group 3 patients (mCRPC-ART) had more advanced disease at baseline compared to groups 1 (mCNPC) and 2 (mCRPC). Group 1 was small due to a recent trend towards abiraterone treatment in this setting and patients had a very high-volume disease that required intensive treatment. In addition, scanning intervals were not always uniformly assessed and confirmatory scans were not conducted in general.

## Conclusion

This study represents the strongest evidence to date that FT plays a fundamental role during docetaxel chemotherapy. In mCNPC and mCRPC patients, complete FT suppression (= 100%) during chemotherapy was an independent predictor of PSAR, RR, PFS and OS. However, in mCRPC patients with a history of ART, FT was not linked to the clinical outcome. Our data suggest that castration-dependent pharmacokinetics of docetaxel seem to reduce its clinical effectiveness in mCRPC-ART patients.

## References

[1] Crawford ED (2019). Androgen-targeted therapy in men with prostate cancer: Evolving practice and future considerations. Prostate Cancer Prostatic Dis..

[2] Teo MY, Rathkopf DE, Kantoff P (2019). Treatment of advanced prostate cancer. Annu Rev Med..

[3] Sartor O, de Bono JS (2018). Metastatic prostate cancer. N. Engl. J. Med..

[4] Scher HI (2008). Design and end points of clinical trials for patients with progressive prostate cancer and castrate levels of testosterone: Recommendations of the Prostate Cancer Clinical Trials Working Group. J. Clin. Oncol..

[5] Ryan CJ (2020). Androgen decline and survival during docetaxel therapy in metastatic castration resistant prostate cancer (mCRPC). Prostate Cancer Prostatic Dis..

[6] Ryan CJ (2013). Serum androgens as prognostic biomarkers in castration-resistant prostate cancer: Results from an analysis of a randomized phase III trial. J. Clin. Oncol..

[7] Diver M (2009). Laboratory measurement of testosterone. Front. Horm. Res..

[8] Morote J (2005). Behavior of free testosterone in patients with prostate cancer on androgen deprivation therapy. Int. J. Biol. Mark..

[9] Rove KO (2014). Maximal testosterone suppression in prostate cancer—Free vs total testosterone. Urology.

[10] von Klot CA (2017). Role of free testosterone levels in patients with metastatic castration-resistant prostate cancer receiving second-line therapy. Oncol. Lett..

[11] Delanoy N (2018). Sequencing of taxanes and new androgen-targeted therapies in metastatic castration-resistant prostate cancer: Results of the international multicentre retrospective CATS database. Eur. Urol. Oncol..

[12] Mezynski J (2012). Antitumour activity of docetaxel following treatment with the CYP17A1 inhibitor abiraterone: Clinical evidence for cross-resistance?. Ann. Oncol..

[13] Lolli C (2019). Testosterone levels and androgen receptor copy number variations in castration-resistant prostate cancer treated with abiraterone or enzalutamide. Prostate.

[14] Gan L (2009). Inhibition of the androgen receptor as a novel mechanism of taxol chemotherapy in prostate cancer. Cancer Res..

[15] Montgomery RB (2008). Maintenance of intratumoral androgens in metastatic prostate cancer: A mechanism for castration-resistant tumor growth. Cancer Res..

[16] Halabi S (2014). Updated prognostic model for predicting overall survival in first-line chemotherapy for patients with metastatic castration-resistant prostate cancer. J. Clin. Oncol..

[17] Scher HI (2016). Trial design and objectives for castration-resistant prostate cancer: Updated recommendations from the Prostate Cancer Clinical Trials Working Group 3. J Clin Oncol..

[18] Eisenhauer EA (1990). New response evaluation criteria in solid tumours: Revised RECIST guideline (version 1.1). Eur. J. Cancer.

[19] Bellera CA (2010). Variables with time-varying effects and the Cox model: Some statistical concepts illustrated with a prognostic factor study in breast cancer. BMC Med. Res. Methodol..

[20] Giobbie-Hurder A, Gelber RD, Regan MM (2013). Challenges of guarantee-time bias. J. Clin. Oncol..

[21] de Bono JS (2017). Subsequent chemotherapy and treatment patterns after abiraterone acetate in patients with metastatic castration-resistant prostate cancer: Post hoc analysis of COU-AA-302. Eur. Urol..

[22] Schweizer MT (2014). The influence of prior abiraterone treatment on the clinical activity of docetaxel in men with metastatic castration-resistant prostate cancer. Eur. Urol..

[23] Tannock I (1989). Treatment of metastatic prostatic cancer with low-dose prednisone: Evaluation of pain and quality of life as pragmatic indices of response. J. Clin. Oncol..

[24] Amato RJ, Ellerhorst J, Finn L, Logothetis CJ (1996). Absence of antitumor activity with prednisone in patients with progressive androgen-independent prostate carcinoma. Urol. Oncol..

[25] Marre F (1996). Hepatic biotransformation of docetaxel (Taxotere) in vitro: Involvement of the CYP3A subfamily in humans. Cancer Res..

[26] Kawano S, Kamataki T, Yasumori T, Yamazoe Y, Kato R (1987). Purification of human liver cytochrome P-450 catalyzing testosterone 6 beta-hydroxylation. J. Biochem. (Tokyo).

[27] Yamazaki H, Shimada T (1997). Progesterone and testosterone hydroxylation by cytochromes P450 2C19, 2C9, and 3A4 in human liver microsomes. Arch. Biochem. Biophys..

[28] Nallani SC, Goodwin B, Buckley AR, Buckley DJ, Desai PB (2004). Differences in the induction of cytochrome P450 3A4 by taxane anticancer drugs, docetaxel and paclitaxel, assessed employing primary human hepatocytes. Cancer Chemother. Pharmacol..

[29] Franke RM, Carducci MA, Rudek MA, Baker SD, Sparreboom A (2010). Castration-dependent pharmacokinetics of docetaxel in patients with prostate cancer. J. Clin. Oncol..

[30] Gillessen S (2020). Management of patients with advanced prostate cancer: Report of the advanced prostate cancer consensus conference 2019. Eur. Urol..

